# The double whammy of ER-retention and dominant-negative effects in numerous autosomal dominant diseases: significance in disease mechanisms and therapy

**DOI:** 10.1186/s12929-024-01054-1

**Published:** 2024-06-27

**Authors:** Nesrin Gariballa, Feda Mohamed, Sally Badawi, Bassam R. Ali

**Affiliations:** 1 Department of Genetics and Genomics, College of Medicine and Health Sciences, United Arab Emirates University, P.O. Box: 15551, Al-Ain, United Arab Emirates; 2 ASPIRE Precision Medicine Research Institute Abu Dhabi, United Arab Emirates University, Abu Dhabi, United Arab Emirates

**Keywords:** ERAD, Dominant-negative effects, Misfolded proteins, ER-retention, Heteromeric complexes

## Abstract

The endoplasmic reticulum (ER) employs stringent quality control mechanisms to ensure the integrity of protein folding, allowing only properly folded, processed and assembled proteins to exit the ER and reach their functional destinations. Mutant proteins unable to attain their correct tertiary conformation or form complexes with their partners are retained in the ER and subsequently degraded through ER-associated protein degradation (ERAD) and associated mechanisms. ER retention contributes to a spectrum of monogenic diseases with diverse modes of inheritance and molecular mechanisms. In autosomal dominant diseases, when mutant proteins get retained in the ER, they can interact with their wild-type counterparts. This interaction may lead to the formation of mixed dimers or aberrant complexes, disrupting their normal trafficking and function in a dominant-negative manner. The combination of ER retention and dominant-negative effects has been frequently documented to cause a significant loss of functional proteins, thereby exacerbating disease severity. This review aims to examine existing literature and provide insights into the impact of dominant-negative effects exerted by mutant proteins retained in the ER in a range of autosomal dominant diseases including skeletal and connective tissue disorders, vascular disorders, neurological disorders, eye disorders and serpinopathies. Most crucially, we aim to emphasize the importance of this area of research, offering substantial potential for understanding the factors influencing phenotypic variability associated with genetic variants. Furthermore, we highlight current and prospective therapeutic approaches targeted at ameliorating the effects of mutations exhibiting dominant-negative effects. These approaches encompass experimental studies exploring treatments and their translation into clinical practice.

## Background

The exploration of molecular and cellular mechanisms underlying many genetic diseases typically starts by evaluating the early stages of the protein biogenesis as well as its subsequent processes including trafficking, interactions, the execution of its biological function and even its disposal. A detailed understanding of defects and aberrations in these processes provides key insights into the molecular foundation of the pathogenesis of genetic diseases and the consequent manifestations of the pathological phenotypes. In eukaryotic cells, secretory and endomembrane proteins destined for many cellular organelles typically enter the endoplasmic reticulum (ER) in their unfolded states, where they undergo their initial and crucial processes to acquire their proper tertiary conformations [1, 2]. In particular, this is where these ER-targeted proteins undergo essential post-translational modifications including glycosylation, proline isomerization, lipidation and disulfide bond formation, which are often crucial for guiding proper folding, stability and the performance of their biological functions [3, 4]. To ensure efficiency and fidelity, cells have adapted extensive ER quality control (ERQC) mechanisms that allow only properly folded proteins to reach their functional destination [5]. Due to the extensive and rigorous cellular mechanisms dedicated to maintaining protein fidelity and proper conformation, it is estimated that 12–15% of newly synthesized proteins do not successfully attain their intended conformation, leading to their subsequent elimination via one or more of the cellular degradation pathways within the secretory pathway [6, 7]. This percentage is significantly increased when proteins harbor mutations that lead to their mis- or mal-folding, and often the removal of the mutant protein quantitatively [8]. Disease-causing mutations, including point mutations, insertions, deletions and repeat expansions impact protein structure and function in diverse ways, leading to three primary disease cellular mechanisms: loss-of-function, gain-of-function or dominant-negative effects [9]. Loss-of-function mutations may cause decreased or total loss of protein function that consequently leads to failure or reduction in performing its normal physiological function [10]. Conversely, gain-of-function mutations occur when the mutant protein acquires a new or abnormal function such as increased or uncontrolled activities leading to dysregulation in the normal cellular activities [11]. In autosomal dominant diseases where one allele expresses the mutant protein, while the other allele preserves its WT expression, a range of disease mechanisms can be demonstrated including loss-of-function of one allele (haploinsufficiency), gain-of-function, dominant-negative effects or a combination of two mechanisms [11]. The dominant-negative effects exerted by the mutant protein on the WT protein may exacerbate the haploinsufficiency state in autosomal dominant diseases [12]. This occurs through interference with the function or trafficking of the normally functioning protein. It is common for proteins to function as homodimers, oligomers or part of multi-subunit complexes. As a consequence, when a mutant protein is expressed from the mutant allele, this can lead to the formation of abnormal dimers, heteromers or multi-subunit complexes that often negatively impact the function or stability of their functioning unit. Numerous studies, including our own, indicate that ER-targeted mutant proteins that are unable to attain their correct conformation, often experience defective trafficking, leading to their entrapment in the ER and subsequent degradation via the ER-associated protein degradation (ERAD) and other associated mechanisms [13–20]. Therefore, exploring whether certain ER-retained mutant proteins exert dominant-negative effects on their WT counterparts, or their complexes has become crucial. Such effects may exacerbate the disease pathological state, potentially explaining some of the broad and variable spectrum of phenotypic expressivity observed for many monogenic diseases [12]. Despite extensive research efforts directed towards comprehending loss of function and gain of function mutations in dominant conditions, there has been relatively limited exploration into the involvement of dominant-negative effects mechanisms. ERAD is a complex mechanism that plays a crucial role in protein quality control in the secretory pathway, involving the recognition of mutant, orphaned and misfolded proteins then targeting them for re-translocation for degradation by the proteasomal or lysosomal machineries. This mechanism has been implicated in the pathology of numerus human genetic condition and the number is expected to keep rising due to the central importance of this quality control mechanism in the monitoring almost a third of the cellular proteins [21–23].

In this manuscript, we aim to review the literature and include our perspectives on the impact of various dominant-negative effects implicated in the pathology of a spectrum of autosomal dominant diseases caused by mutations in secretory and membrane proteins (Table 1). We will focus on both the currently acknowledged and potential dominant-negative effects displayed by mutant variants retained in the ER as a result of the ER quality control (ERQC) mechanisms. In addition, we will also highlight existing and potential therapeutic interventions aimed at mitigating the impact of mutations exhibiting dominant-negative effects, spanning from experimental research on therapies to their application in patient care.

**Table 1:** Autosomal dominant monogenic diseases caused by mutant secretory and membranal proteins

Genetic Disorder	Gene	Protein formation (Dimer/Oligomer)	Reported ER retention	Reported DN effect	Therapeutic strategies overcoming DN effects and cellular consequences
**Skeletal and Connective Tissues Disorders**					
Marfan Syndrome (MFS)	*FBN1*	Dimer	[24]	[25]	Gene editing [26] TGF-β antagonists to block the excessive TGF-β signaling [27]
Idiopathic short stature syndrome	*NPR2*	Dimer	[28]	[28–32]	Recombinant growth hormone replacement therapy [33]
Limb-girdle Muscular Dystrophy (LGMD-1C)	*CAV3*	Oligomer	[34]	[34]	Gene therapy: recombinant AAV1 vector-based therapy for various LGMD-related gene (Sarcoglycan, DYSF, and SGCB) stimulators [35] Small molecule correctors: lumacaftor and tezacaftor [36]
Bethlem Myopathy Disorder	*COL6A1, COL6A2* and *COL6A3*	Heterotrimer	NR	[37]	Molecular modulators: cyclosporin A rescues the mitochondrial dysfunction and decreases apoptosis [38]
Osteogenesis Imperfecta	*COL1A1* and *COL1A2*	Homotrimer	[39, 40]	[41]	Signaling pathway modulators: Denosumab, Romosozumab, and Anti-TGF-β Antibodies [42] Bone marrow and stem cell transplantation [42] Gene therapy via antisense oligodeoxyribonucleotides; siRNA, short interfering RNA; CRISPR–Cas9 and hammerhead ribozymes [42] Counteraction of ER Stress and UPR (4-phenylbutyrate) [42]
Ehlers-Danlos Syndrome	*COL5A1* and *COL5A2*	Heterotrimer	NR	[43]	Gene therapy: Allele-specific siRNA knockdown [44] Signaling pathway modulators: Celiprolol (cardioselective β-blocker) [44]
Stickler syndrome	*COL2A1, COL11A1* and *COL11A2*	Heterotrimer	[45]	[46]	NR
**Vascular Disorders**					
Hereditary Haemorrhagic telangiectasia type 1 (HHT1)	*ENG*	Dimer	[13]	[47]	ENG and ACVRL1 gene expression stimulator [48]
Hereditary Haemorrhagic telangiectasia type 2 (HHT2)	*ACVRL1*	Dimer	[16]	[49]	FGF signaling modulation via beta-blockers: Etamsylate [48] Angiogenesis signaling pathways modulation: Bevacizumab, thalidomide, nintedanib, and anti-ANGPT2 antibodies [48]
Pulmonary arterial hypertension (PAH)	*BMPR2*	Dimer	[15]	[50]	Counteraction of ER Stress and UPR (4-phenylbutyrate) [50] Targeting various PAH molecular pathways: 1) endothelin receptor signaling, 2) nitric oxide-sGC signaling, 3) prostacyclin replacement or receptor agonists, and 4) calcium channel blockers [51]
Loeys-Dietz Syndrome (LDS)	*TGFBR1, TGFBR2, SMAD2, SMAD3, TGFB2* and* TGFB3*	Dimers	NR	[52]	Gene editing: correcting genetic mutations in TGFBR1 gene via CRISPR-Cas9 in human-derived iPSC [53]
Long QT syndrome (LQTS) type 2	*KCNH2*	Dimer	[54]	[54]	Gene therapy: single suppression-replacement of *KCNH2* gene therapy using shRNA in iPSC- patient-derived cardiomyocyte [55] β-blocker medications [56] Pharmacological correction of the trafficking defect and ER retention using chaperones and channel blockers [57]
**Neurological Disorders**					
Generalized epilepsy with febrile seizures (GEFS +)	*GABRG2*	Pentamer	[58]	[59]	Gene therapy; γ2 subunit gene (GABRG2) replacement therapy [58]
spinocerebellar ataxias 13	*KCNC3*	Tetramer	[60]	[60]	Trafficking defect correction: Co-expression of the epidermal growth factor receptor (Egfr) with the DM KCNC3^R423H^ effectively rescues the eye developmental defects (Drosophila model) [60]
Neurofibromatosis Type 1 (NF1)	*NF1*	Dimer	[61]	[61]	Gene therapy: 1) Nonsense suppression using Aminoglycoside antibiotics to cause a read-through of nonsense mutations and restore the functional protein in short-term studies 2) Splice-blocking antisense oligonucleotides (ASOs) can effectively skip mutant exons in cultured cells with NF1 deep intronic mutations, restoring neurofibromin expression 3) recombinant rAAV carrying the WT *NF1* [62] Targeting ER stress through a combination treatment of Hsp90 inhibitor and rapamycin immunosuppressant [62]
DYT1 dystonia	*TOR1A*	oligomer	[63, 64]	[63, 64]	Gene therapy: 1) Allele-specific targeting of mutant TOR1A by the compact CRISPR/NmCas9 system 2) Gene editing via CRISPR/Cas9 to repair the mutation site in the DYT- TOR1A gene and restore its normal function [65]
**Eye Disorders**					
Retinitis Pigmentosa (RP)	*RHO*	Dimer/Oligomer	[66]	[67–69]	Blocking the gene product from the mutant allele through ribozymes [70] Posttranslational gene silencing via shRNA and RNAi [70]
Primary open angle glaucoma (POAG)	*MYOC*	Oligomer	[71]	[72]	Gene therapy: 1) knocking out *MYOC* via viral-mediated CRISPR/Cas9 [73] Inhibiting *MYOC* mRNA transcription or translation through siRNA and shRNA [73] Using chemical chaperones to reduce protein misfolding and increase mutant myocilin secretion [74]
Wolfram syndrome (WS)	*WFS1*	Monomer (WT) Aggregate (Mutant)	[75]	[75]	Gene therapy: mutant allele replacement via CRISPR/Cas9 in patient iPSCs to create iPSC-derived organoids [76] Correct protein misfolding and stabilization: chemical and molecular chaperones: 4-phenylbutyric acid, tauroursodeoxycholic acid and sigma-1 receptor chaperone [76] Regulating ER calcium homeostasis using ER calcium stabilizers: Ibudilast and dantrolene [76] Targeting ER stress: Valproic acid and GLP-1R agonist like liraglutide [77]
**Serpinopathies**					
Antithrombin deficiency	*SERPINC1*	Monomer (WT) Aggregate (Mutant)	[78]	[78]	NR
Alpha-1-antitrypsin deficiency (AATD)	*SERPINA1*	Monomer (WT) Aggregate (Mutant)	[79]	[79]	Gene therapy: 1) Supplementation of the WT gene through viral transduction in fibroblasts derived from patients [80] 2) liver-directed rAAV-mediated gene augmentation [80] 3) mRNA silencing via specific RNAi (Fazirsiran) [81]
Hereditary angioedema type 1 (HAE1)	*SERPING1*	Monomer (WT) Aggregate (Mutant)	[81]	[81]	Gene therapy: WT *SERPING1* gene supplementation via AAV vector [82]

The table highlights the reported dominant-negative effects and ER-retention implicated in the molecular mechanisms of the listed diseases. It also highlights reported therapeutic strategies overcoming dominant negative effects and cellular consequences

*NR* Not reported, *DN* Dominant-negative

## The dominant-negative effects: Concept and mechanisms

Dominant-negative mutations have been defined in 1987 by Ira Herskovitz as “those leading to mutant polypeptides that disrupt the activity of the WT gene when overexpressed” [83, 84]. This current review is focused on the combinational mechanism of ER retention and dominant-negative effects exerted by the mutant proteins on their WT counterpart, resulting in the entrapment and defective trafficking of WT proteins from the ER to their functional destinations. `It is crucial, however, to recognize that dominant-negative mechanisms extend far beyond those caused by ER-retained mutants. Here is an overview of some of these mechanisms:

1. Altered protein trafficking: Mutant proteins might alter the trafficking of the WT proteins causing their entrapment in cellular compartments, thus preventing their trafficking to their functional destination. For example, some ER-retained endoglin mutants associated with hereditary haemorrhagic telangiectasia type 1(HHT1) form heterodimers with WT endoglin in the ER and thus preventing its trafficking to the plasma membrane [47]
2. The formation of inactive protein complexes: Trafficking-competent mutant proteins can form heterodimers with their WT counterpart expressed by the unaffected allele or with other WT partner proteins expressed by different genes, which renders the heterodimeric complex inactive [85]. This type of dominant-negative effect is best represented in collagen disorders such as Osteogenesis Imperfecta (OI), in which a mutant collagen protein forms inactive complexes with its WT partners negatively impacting the function of the whole collagen matrix [39].
3. Competitive binding inhibition: In this case, the mutant proteins compete with the WT counterparts for binding to shared substrates or ligands, thereby limiting proper binding interactions, leading to a potential inhibitory effect. For example, Von Willebrand disease (VWD) is a bleeding disease caused by mutants von Willebrand factor (VWF), a glycoprotein expressed by endothelial cells [86]. Mutants VWF interfere with the binding of WT protein to platelets and sub-endothelium in a dominant-negative manner, resulting in reduced clotting function and increased risk of bleeding [87].
4. Protein destabilizing effect: The dominant-negative effect exerted by the mutant protein results in WT reduced stability or even premature degradation. Certain p53 tumor suppressor mutant proteins adversely affect the stability of WT p53, leading to its reduced function and effectiveness [88].
5. Conformational effects: Secreted misfolded mutant proteins with structural defects may give rise to conformational defects in the WT protein complex, impeding its physiological function. For example, mutant fibrillin-1 proteins encoded by FBN1 gene, associated with Marfan disease exhibit a dominant-negative effect on the WT protein via disruption of the normal assembly of the extracellular matrix [89].

## Protein quality control mechanisms in the early secretory pathway: Components and involvement in human diseases

**Fig. 1:**
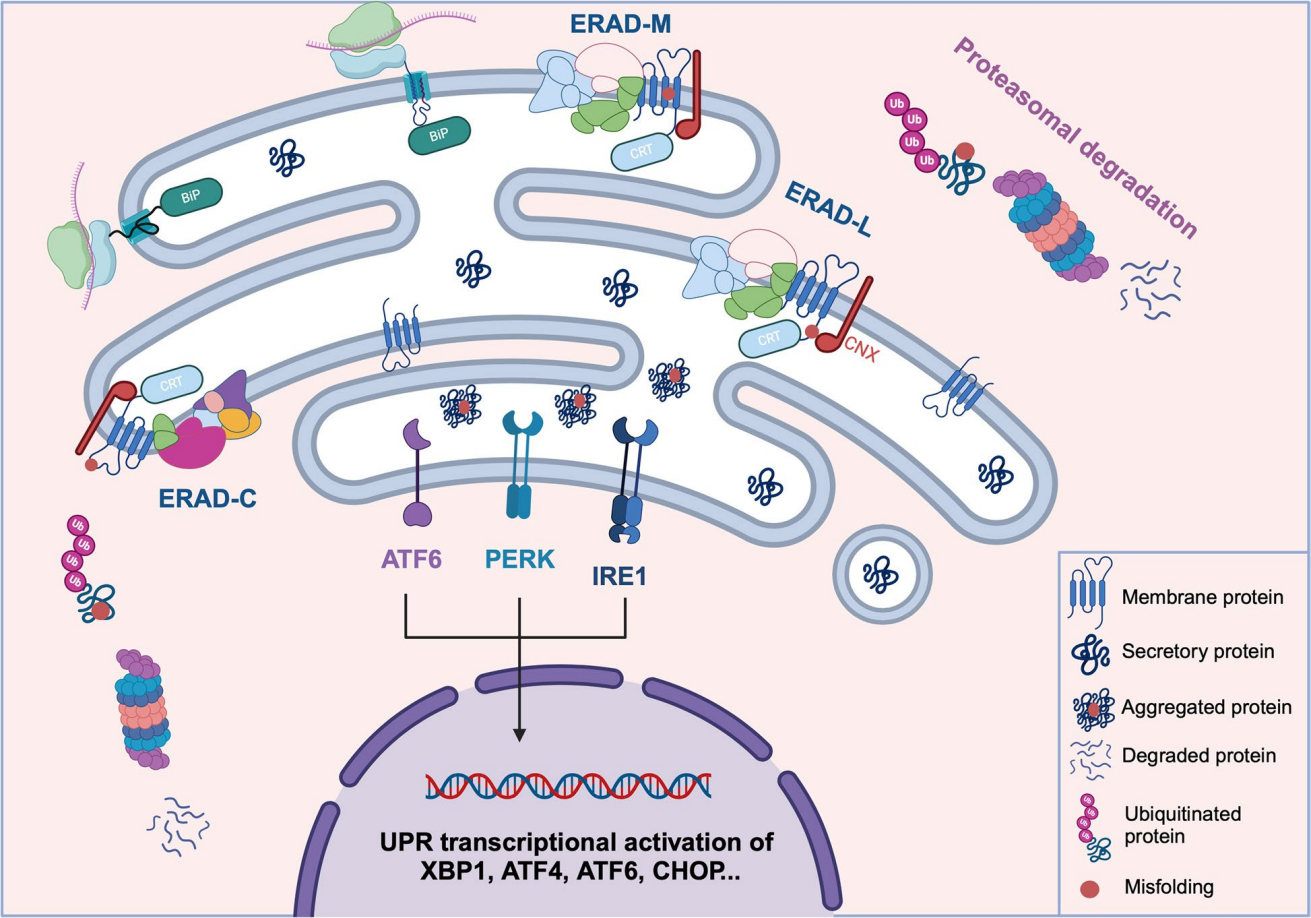
Protein synthesis and quality control mechanisms in the ER. Newly synthesized proteins in the ER undergo quality control checks to ensure correct folding. Misfolded proteins form aggregates in the ER lumen and activate the UPR response that leads to transcriptional activation of various ER stress genes and are degraded via the proteasomal ERAD machinery based on their lesioned domain

Newly synthesized secretory and membrane proteins are transported to the ER where they undergo various interactions, posttranslational modifications and assembly to generate mature proteins in fully folded three-dimensional states. While protein folding can occur co- or posttranslationally, its accuracy and efficiency are important factors for the protein’s functionality and cellular homeostasis. Protein folding in the ER is mediated via a complex ER-resident chaperoning and folding machineries, consisting of the heat shock protein (Hsp) chaperones and the calnexin/calreticulin (CNX/CRT) lectin-like chaperones [90]. Among the major molecular chaperones involved in preventing protein aggregations and incorrect folding is the Hsp70 chaperone; BiP (Fig. 1) [91, 92]. Dysfunction in the chaperoning machinery contributes to various diseases, including Alzheimer's and Parkinson diseases [93].

In addition to the failure of the chaperoning systems, several other factors contribute to defective protein folding in the ER, including transcriptional or translational errors, abnormal chemical protein modifications, oxidative stress, and genetic mutations [18, 94]. Consequently, incorrect protein folding results in defective trafficking of the proteins to their destination associated with ER retention and the formation of protein aggregates in the ER lumen. Accumulation of misfolded proteins in the ER triggers an ER stress signaling cascade known as the unfolded protein response (UPR). The UPR employs cytoprotective strategies aimed at preserving ER homeostasis, thereby alleviating ER stress caused by the burden of misfolded proteins. This is accomplished by activating downstream ER quality control activities. The UPR cascade is induced by three established arms: protein kinase RNA like endoplasmic reticulum kinase (PERK), inositol requiring enzyme 1 (IRE1), and activating transcription factor 6 (ATF6) (Fig. 1) [20, 95]. The coordinated activation of the three UPR arms collectively targets the attenuation of protein synthesis within the ER, induction of molecular chaperones’ gene expression aiding in protein folding, and ultimately the removal of misfolded proteins via ERAD mechanism [96]. ER stress and the disruption of the UPR have been linked to the development of various diseases, spanning neurodegenerative conditions like Alzheimer's and Parkinson's diseases [97], metabolic disorders such as diabetes and obesity [98–100], inflammatory diseases [101], cancer [102, 103], and rare genetic disorders like cystic fibrosis (CF) [104], Gaucher disease [105, 106], Acromesomelic Dysplasia 1, Maroteaux type [18] and many others [107–110]. Understanding the mechanisms underlying ER stress and the UPR activation is crucial for elucidating disease pathogenesis and developing therapeutic strategies. CF has been recognized as a pioneering disease in the field of ER stress research due to its well-defined genetic basis, thoroughly investigated pathophysiology, and the availability of animal models and cell culture systems for studying disease mechanisms [111]. CF is an autosomal recessive disorder caused by mutations in the CFTR (cystic fibrosis transmembrane conductance regulator) gene, which encodes a chloride channel primarily found in the apical membrane of epithelial cells [112]. Mutations in CFTR result in defective chloride ion transport across cell membranes, leading to sticky mucus production in the lungs and digestive system. Studies have revealed that most CF disease-causing mutations, including the most common CF variant (F508del), result in defective protein folding and trafficking, leading to ER retention and degradation of misfolded CFTR protein [113–117]. This accumulation of misfolded CFTR protein in the ER triggers ER stress and activates the the UPR mechanism aimed at restoring ER homeostasis [118].

In eukaryotic cells, nascently synthesized proteins are subjected to unique quality control assessments. This step involves the UDP-glucose: glycoprotein glucosyltransferase (UGGT) interaction with partially or misfolded proteins providing a further folding attempt mediated by the CNX/CRT chaperones cycle [119]. Proteins failing to meet the stringent quality checks are destined for degradation via various pathways including the ERAD machinery. Misfolded proteins are recognized and processed by distinct ERAD sub-pathways: ERAD-L, ERAD-M, and ERAD-C, based on the site of the defective domain within the protein—luminal (ERAD-L), membrane-bound (ERAD-M), or cytosolic (ERAD-C) (Fig. 1) [120]. ERAD substrates are exported and tagged with ubiquitin that serves as a degradation signal. These proteins are retrotranslocated to the cytosol for degradation by the large protein complex, the proteasome or the lysosomes, via a pathway known as the ER-to-lysosome associated degradation (ERLAD) [121]. Moreover, some ER retained mutants might escape the ERQC mechanism and avoid being degraded. These mutants might form aggregates in the ER lumen or interact in a dominant-negative manner with their WT counterparts and possibly affect their normal physiological function. These mechanisms and indeed defects in their components have been implicated in numerous human conditions, which is the focus of this review.

## ER-retained mutant proteins exhibit dominant-negative effects in a range of autosomal dominant disorders

**Fig. 2:**
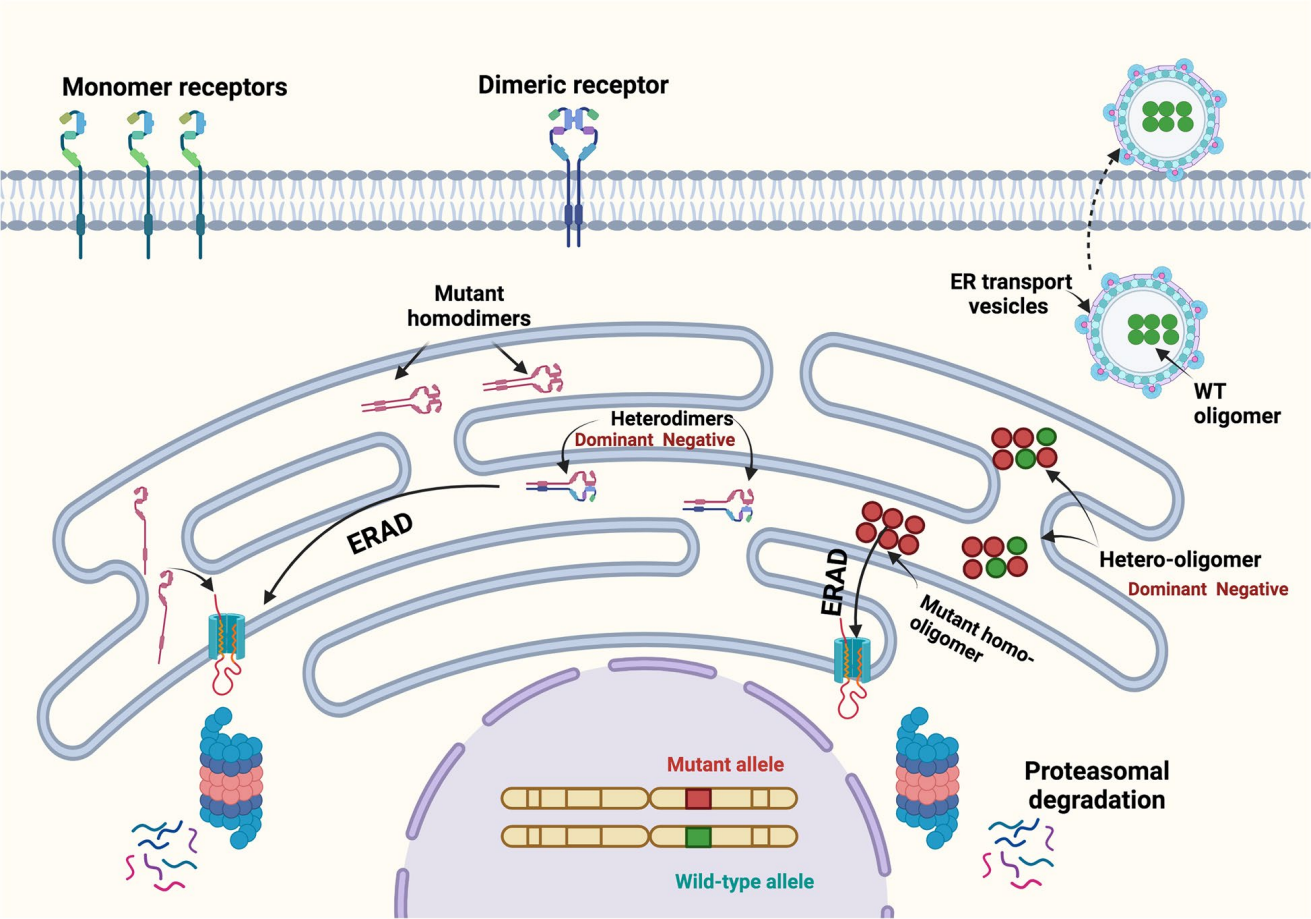
Dominant-negative effects exerted by ER-retained mutant on the WT protein expressed by the functional allele. Monomeric Plasma membrane proteins or secretory proteins that fail to attain their normal conformation get targeted by the ERAD machinery for proteasomal degradation. In the case of dimeric or oligomeric proteins, ER-retained mutants are likely to interfere with the WT counterpart via a formation of hetero dimers/oligomers complexes. This interreference causes the entrapment of the WT within these complexes impeding its normal trafficking and leading to premature degradation through the ERAD mechanism

As indicated earlier, the entrapment of secretory proteins may result in a spectrum of monogenic diseases with varied modes of inheritance and molecular mechanisms. These encompass a range from loss of function, gain of function, dominant-negative effects, or combinations thereof. Here, we emphasize the dominant-negative effects demonstrated or predicted to play significant roles in the pathology of numerous autosomal dominant diseases. In these conditions, both the WT and mutant variants are expressed and anticipated to interact during the initial stages of the protein biogenesis and assembly within the ER, particularly in cases where the protein functions as a dimer, oligomer or part of a multi-subunit complex (Fig. 2). The broad spectrum of phenotypic severity observed across various autosomal dominant diseases constitutes a significant area of ambiguity and concern in biomedical research. The exploration of mechanisms, or combinations thereof, that cause phenotypic heterogeneity among affected individuals is essential in understanding the complex genotype–phenotype interplay. Extensive literature highlights examples of dominant-negative effects instigated by mutant variants, wherein these mutants impair the function of the WT counterpart at the functional location. In this review, we specifically explore the dominant-negative effects exerted by ER-retained mutants on the WT proteins, thereby initiating a double effect that entails dominant-negative effects on top of haploinsufficiency leading to the entrapment and possibly premature degradation of WT protein. Consequently, this combinatorial mechanism causes an excessive loss of protein function, thereby exacerbating the severity of disease phenotypes for some mutants exhibiting ER retention. It is important to note that our aim in this review is not to exhaustively cover every condition exhibiting dominant-negative effects within the field, but rather to highlight the widespread involvement of these mechanisms in disease pathogenesis in some autosomal dominant conditions.

### Skeletal and connective tissue disorders

Marfan Syndrome (MFS, MIM #154700) is an autosomal dominant genetic disease caused by heterozygous mutations in the *FBN1* gene that encodes Fibrillin-1, a crucial protein component in the extracellular matrix (ECM) [122]. The disease is broadly classified as a connective tissue disorder with clinical manifestations impacting various organs including the heart, the blood vessels and the eyes [123]. The most distinctives features include a long face, high-arched palate, elongated limbs, tall and slender physique, chest deformities, lens dislocation, aortic root dilation and potential aneurysms [122]. MFS is characterized by a wide spectrum of phenotypic variabilities among affected individuals, including those carrying different genetic variants or even those with the same variant [123]. Fibrillin family of proteins encompasses three main types of fibrillin, fibrillin-1, 2 and 3. Fibrillin-2 and 3 are predominantly expressed during development, whereas fbrillin-1 is expressed throughout adulthood, as it provides strength and elasticity to connective tissues in major organs [124]. Fibrillin-1 is a 250 kDa glycoprotein characterized by multi-modular organization and is considered as the major structural component of the connective tissues microfibrils [24]. However, the mechanism by which fibrillin-1 assembles into microfibrils, remains to be fully elucidated. The majority of disease-causing variants in *FBN-1* involve the substitution of cysteine amino acids, which play a critical role in forming disulphide bridges and maintaining proper protein conformation. For instance, severely misfolded variants like C1117Y and C1129Y, where cysteine is replaced by tyrosine, exhibit defective trafficking and become trapped and accumulate in the ER [24]. Conversely, the disease-causing variant G1127S is secreted normally, similar to the WT. These conclusions were drawn by examining their glycosylation profiles. Variants C1117Y and C1129Y exhibit a simple N-linked glycosylation pattern characteristic of ER acquisition. On the other hand, they lack the complex N-glycosylation typical of Golgi apparatus processing, resulting in a lower molecular weight presentation on SDS-PAGE, confirming their retention in the ER. In contrast, variant G1127S appears in a mature, fully glycosylated form, mirroring WT fibrillin-1 [24]. As a result, it was proposed that normally-trafficked fibrillin-1 mutants such as G1127S are likely to exert dominant-negative effects via misincorporation into the normal microfibril, however, no clear evidence was presented. In addition, intracellular dominant-negative was postulated as a probable disease mechanism attributed to the ER-retained variants such as C1117Y and C1129Y in addition to haploinsufficiency. This occurs because ER-retained mutants are prone to interact with WT fibrillin-1, forming heterodimers during the initial stages of protein dimerization within the ER. This interaction hampers the trafficking and secretion of fibrillin-1 to the cell surface [24]. The concept of a dominant-negative effect was previously proposed by Dietz and colleagues*,* supported by their demonstration that patients expressing the least amount of the nonsense mutant variants of fibrillin-1 exhibited the mildest disease phenotype. Conversely, patients with fully expressed variants exhibited the usual moderate to severe manifestation of the diseases [25].

Recent advances in high-throughput genomic analysis have revealed that idiopathic short stature (ISS, MIM # 300582) in children is linked to various genes that regulate growth plate function, including heterozygous mutations in *NPR2* [29]. NPR-B encoded by *NPR2* functions as a homodimer that catalyzes the conversion of GTP to cGMP upon binding of its ligand, C-type natriuretic peptide (CNP) [30]. Recently, co-immunoprecipitation assays have revealed that diseases-causing heterozygous missense NPR-B variants (R110C, R495C and Y598N) identified in ISS subjects exhibit dominant-negative effects on the WT receptor [29, 31]. These studies have demonstrated that intracellular cGMP levels significantly increase upon cell transfection with the WT NPR-B. On the other hand, a significant decrease is observed when a mutant variant is co-expressed with the WT receptor. Notably, a prior investigation has also identified variant R110C as an ISS-causing mutation. This study demonstrated that this particular variant displays defective trafficking from the ER to the Golgi apparatus, evidenced by an immature glycosylation profile characteristic of ER-retained mutants [28]. Conversely, variant Q417E, also identified in the same study, trafficked normally to the plasma membrane. Interestingly, both variants exhibited dominant-negative effects, as evidenced by a decrease in cGMP production capacities. Specifically, R110C showed a negligible cGMP response, while Q417E displayed a significantly reduced response. These findings underscore the importance of further investigating the implications of dominant-negative effects and ER retention across a broader spectrum of variants. This approach will enable researchers to gain a more comprehensive understanding of the molecular pathology associated with variable variants.

Autosomal dominant Limb-girdle Muscular Dystrophy (LGMD-1CI, MIM # 609115) is a specific subtype of LGMD that is associated with mutations in *CAV3* gene. The disorder is characterized by progressive muscle weakness primarily affecting the shoulders and hips muscles [125]. Caveolin-3 encoded by *CAV3* is a member of the caveolin integral membrane proteins and a key structural protein of caveolar membrane in muscle cells [126]. Up to date ten LGMD-1C-disease causing variants of *CAV3* have been identified according to the Human Genome Mutation Database (HGMD) (https://www.hgmd.cf.ac.uk/ac/index.php). Amongst the most studied variants are P104L, ΔTFT/63–65 (deletion of amino acids 63 to 65) and A45T. Minetti, Sotgia, Lisanti, and colleagues were the first to report the disease causing variants P104L, ΔTFT/63–65 in LGMD-1C patients [127]. In vitro characterization and immunofluorescence staining experiments have shown that the two variants fail to reach the plasma membrane. Instead, they are retained at the Golgi complex level and subsequently degraded through proteasomal degradation, along with partial degradation of the WT protein [128]. These findings suggest a dominant-negative effect of these two variants on the WT protein, leading to its entrapment in mixed WT/mutant oligomers, which ultimately results in proteasomal degradation, as evidenced by ER-localization. The same research group later demonstrated that treatment with the proteasomal inhibitor (MG-132) significantly enhanced the trafficking of WT protein entrapped at the Golgi complex to the plasma membrane in cells expressing both the WT and mutant variants [34]. Similarly Herrmann and colleagues have demonstrated that disease-causing variant A45T exhibit defective trafficking and also prevent the normal localization of WT caveolin-3 in a dominant-negative manner [129].

### Collagen-related mutations in connective tissue disorders

The intricacy of collagen structures and their assembly plays a pivotal role in the diverse phenotypic manifestations observed in individuals with mutant collagen genes. Collagen, being the most abundant protein in the human body and a fundamental component of connective tissues, contributes significantly to the structural integrity of various tissues in the body such as skin, cartilage, and blood vessels [130]. The variability in collagen types and their interactions within heterotypic fibrils adds a layer of complexity to the assembly process. Mutations in collagen genes can disrupt this intricate network, leading to a spectrum of phenotypes broadly known as collagenopathies or connective tissue disorders [131].

A collagen molecule is most characterized by its triple-helical α-domain, which constitutes up to 95% of the molecule in some classes of collagen (reviewed in [132, 133]. Several collagen-related genetic disorders are caused by a dominant inheritance of glycine substitution to a larger amino acid in the triple helical domain of the protein, which structurally affect collagen folding and assembly [131].

Mutations in collagen type VII alpha 1 encoded by the gene *COL7A1* cause dystrophic epidermolysis bullosa (DEB, MIM # 131750), a genetic disorder characterized by skin blistering in response to minor trauma or friction [134]. It has long been established that certain disease-causing variants involving glycine substitutions accumulate intracellularly in the endoplasmic reticulum (ER) and fail to undergo proper extracellular secretion [135]. This failure to secrete results in a haploinsufficiency state, contributing to the manifestation of the disease. The same group has later shown that secreted mutant α1(VII) chains exert a dominant-negative effect by interacting with the WT protein forming heterotrimeric triple helix complex, leading to a destabilizing effect on collagen VII structure [131].

Heterozygous mutations in collagen VI genes (*COL6A1, COL6A2* and *COL6A3*) are associated with Bethlem myopathy disorder (BMD, MIM # 158810), which is a milder form of Ullrich congenital muscular dystrophy (UCMD, MIM # 254090) also associated with homozygous mutations in the same genes. Bethlem myopathy is mainly characterized by proximal muscle weakness and joint contracture that progressively affect mobility and flexibility [136]. However, distinction between the two diseases in terms of their mode of inheritance was revised when patients with heterozygous mutations exhibited a severe form of the disease that is typical of UCMD. This finding has given rise to rigorous protein synthesis studies in order to provide an explanation for the consequences of severe mutations in the extremely complex structure of collagen VI [37, 137]. The three alpha chains α1, α2 and α3 that characterize collagen VI fold into triple helical heterotrimeric monomer in the ER, which are then transported to the cell surface via the Golgi apparatus [133]. When these procollagen monomers reach the cell surface, they align in a staggered fashion to form dimers that are bonded via a disulfide bond, then dimers are aligned laterally to form a tetrameric complex in the extracellular matrix (ECM) [138]. It has been demonstrated that large amino acid deletion at the N terminal of the triple helical domain resulted in the secretion of mutant heterotetramers. The tetrameric stoichiometry of collagen VI means that mutations in any of the three alpha chains would result in only 1/16 normal tetramer [37, 137]. This finding unequivocally illustrates the dominant-negative impact exerted by the mutant chain on the overall structural assembly of collagen VI. As a result, the collective effect resembles the complete loss of collagen VI observed in individuals with the homozygous mutation in UCMD. On the other hand, patients carrying heterozygous, in-frame amino acid deletions downstream of the triple-helical domain, which removes cysteines required for dimerization, exhibit a milder form of the disease. This deletion prevents the formation of WT/mutant dimers and consequently reduces the dominant-negative impact on the WT protein [137].

Osteogenesis imperfecta (OI) has served as a classic example for dominant-negative effects of structural proteins [41]. The disease, also known as brittle bone disease, is characterized mainly by bone fragility, short stature, loose joints and other variable skeletal deformities [139]. Classical OI types I to IV are caused by autosomal inherited mutations in *COL1A1* or *COL1A2* genes that encode α1 and α2 subunits of collagen 1, respectively. Type I collagen is a heterotrimeric complex that consists of two α1 and one α2 chains, synthesized in the ER and assembled via a recognition sequence at the C terminal, along with the formation of disulfide bondings, prior to being transported to the Golgi apparatus [39, 140]. The variability in the phenotype of this disease, coupled with a limited understanding of its underlying mechanisms, has prompted researchers to identify additional collagen-related genes involved in the regulation of collagen metabolism and assembly. Most of these genes have been linked to autosomal recessive pattern of inheritance. Nonetheless, mutations in Collagen1 genes still account for the majority of OI cases [141].

Molecular mechanisms attributed to the manifestation of autosomal dominant OI include decreased transcript due to nonsense mutation, decreased collagen secretion due to ER retention, and disrupted pro-collagen chains assembly and processing [41, 142–144]. ER retention of glycine-substitution mutant variants of α1 and α2 subunits of collagen 1 was observed through transmission electron microscopy analysis of OI fibroblasts, revealing the presence of an enlarged ER indicative of ER stress [145]. Apoptotic cellular death was also demonstrated to be triggered despite autophagic activation through the UPR in an attempt to salvage the cells. Severe forms of OI that involve glycine substitution are associated with pathogenic variants that exert a dominant-negative effect, disrupting the assembly of the triple helix and collagen fibril. This disruption results in severe structural damage to the bone matrix [41, 146, 147].

Ehlers-Danlos syndromes (EDS) represent a group of genetically heterogenous conditions that are caused by pathogenic variants in up to 19 genes, mostly encode collagens or collage-related proteins [148]. Classic EDS (cEDS, MIM # 130000) is mainly associated with mutations in *COL5A1* or *COL5A2* genes encoding α1 and α2 chains of the collagen 5. Patients of this class of EDS present with joint hypermobility, skin hyper-elasticity and a tendency to develop atrophic scars [149]. Despite the limited number of clinically well described cEDS associated with mutations in *COL5A2,* the majority manifest severe phenotypes and have an impact on the structural integrity of collagen V. These mutations exhibit a dominant-negative effects that disrupt the formation of heterotypic fibrils and the interactions between collagen 5 and other constituents of the extracellular matrix [43, 150].

Pathogenic variants of *COL2A1* and *COL11A1* genes encoding collagen II α1 and collagen XI α2 chains have been associated with Stickler syndrome type 1 and 2, (ST1, MIM # 108300 and STL2, MIM # 604841), respectively. The α1 chain from *COL11A1* combines with the α2 chain from *COL11A2* and the α1 chain from *COL2A1* to create heterotrimeric type XI collagen [46]. Stickler syndromes are a group of heterogenous connective tissue disorders characterized by distinctive facial feature, ocular abnormalities and joint anomalies. Splice site mutations in *COL11A2* are the primary disease-causing mutations reported thus far. However, mutant mRNA does not undergo nonsense-mediated decay (NMD), allowing mutant chains to be expressed and associate with other α chains, leading to the formation of mutant collagen XI trimers in a dominant-negative manner [46, 151]. Conversely, patients with mutations that lead to unexpressed protein due to targeting by the NMD mechanism tend to exhibit a milder form of the disease, and this mechanism is considered haploinsufficiency only [152].

Akawi and colleagues have argued that homozygous misfolding mutations in *COL11A* are more severe than bi-allelic null mutations as a result of the possible interference of the misfolded COL11A with its other collagen partners, presumably as a result of dominant-negative effects, and hence disrupting the function of the whole complex more severely [153, 154]. On the other hand, pathogenic variants of *COL2A1*, associated with Stickler syndrome type1, are believed to manifest the disease phenotype through only haploinsufficiency [46]. Several misfolded variants of both types of collagens were reported to be retained in the ER, followed by proteasomal degradation [45]. Nonetheless, to our knowledge, whether ER-retained mutants exert dominant-negative effects by interfering with the WT in heterotrimeric complexes remains largely unexplored.

The preceding discussion highlights how the complexity of collagen structure magnifies the dominant-negative effect of mutant subunits by exacerbating the disruption in the intricate network of collagen assembly. Mutant collagen subunits, with their altered structures, not only compromise the functionality of individual molecules but also introduce a destabilizing influence during fibril formation. These effects ultimately contribute to the diverse phenotypic outcomes observed in collagen-related genetic disorders. It’s important to note that there has been limited research dedicated to investigating the dominant-negative effect exerted by mutant collagen subunits when trapped in the ER, potentially affecting their trafficking. The ER serves as a crucial site for proper folding and post-translational modifications of collagen molecules before they are transported to their functional destinations. The presence of mutant subunits in the ER may disrupt these processes, leading to the accumulation of misfolded or improperly modified collagen. This lack of in-depth exploration into the consequences of such entrapment hinders a comprehensive understanding of how trafficking abnormalities contribute to the overall pathogenesis of collagen-related genetic disorders.

### Vascular monogenic disorders

Hereditary haemorrhagic telangiectasia (HHT) is a vascular genetic disorder characterized by vascular dysplasia inherited in an autosomal dominant manner. Its spectrum of phenotypes varies from occasional nasal bleeds to internal organ hemorrhages affecting the gastrointestinal tracts (GI), kidneys, liver, and brain [155].The disease has been classified into four types according to the causative gene: HHT1, HHT2, HHT5 and (JPH) Juvenile polyposis and HHT, associated with mutations in *ENG, ACVLR1, GDF2* and *SMAD4* genes, respectively [156–159]. These genes encode proteins that are components of the transforming growth factor beta (TGFβ) signaling pathway, which regulates various cellular processes [109, 160, 161]. Hereditary haemorrhagic telangiectasia type 1 (HHT1, MIM # 187300) is associated with mutations in the gene *ENG* that encodes endoglin, a dimeric glycoprotein that functions as a co-receptor on the plasma membrane. It is predominantly expressed in vascular endothelial cells of various tissues and organs throughout the body and it is therefore essential for the normal structure of the blood vessels [162]. In earlier work, we have utilized glycosylation profiling assays and immunofluorescence microscopy to demonstrate that several disease-causing missense endoglin variants get trapped in the ER by the machinery of the ERQC mechanism and fail to traffic to the plasma membrane, where they function [13]. Subsequently, we have also demonstrated the implication of ERAD in the degradation of ER-retained missense mutant variants P165L and V105D using HRD1-knockout HEK293 invitro cellular model [163]. Protein elimination leads to a haploinsufficiency state, ultimately contributing to the manifestation of the disease phenotype. Given that endoglin is a homodimeric protein synthesized in the ER, it was logical to explore whether mutant variants expressed by the affected allele would interact with WT endoglin, potentially leading to a dominant-negative effect. Interestingly, our co-immunoprecipitation assays have clearly demonstrated that ER-retained endoglin variants (L32R, V105D, P165L, I271N and C363Y) heterodimerize with WT endoglin in a dominant-negative manner impairing its trafficking to the plasma membrane [47]. This mechanism is likely to exacerbates the disease state, resulting in a scenario where 50% of the protein is lost due to the loss of one allele leading to haploinsufficiency, coupled with a possible additional ~ 25% loss attributed to the dominant-negative effects exerted by the mutant ER-retained protein on its WT counterpart. Our findings were consistent with two prior studies that briefly illustrated the formation of mixed dimers of endoglin WT and mutant variants [164, 165].

Hereditary haemorrhagic telangiectasia type 2 (HHT2, MIM # 600376) is associated with mutations in *ACVRL1* gene, encoding activin receptor-like kinase, also denoted as ALK1, a type 1 receptor in the TGFβ signaling pathway [166]. Both HHT1 and HHT2 are presented with similar phenotypes, as both endoglin and ALK1 play a crucial role in endothelial cells differentiation during capillary development leading to vascular malformation phenotypes in both disorders [167]. Similar to endoglin, both our research and other studies have shown that several missense mutant variants located at the intracellular kinase domain of ALK1 receptor become entrapped in the ER and fail to traffic to the plasma membrane where they normally perform their functional role in TGFβ signaling cascade [16, 164, 168]. Currently, haploinsufficiency has been accepted as the primary disease mechanism [158, 169]. However, the homodimeric nature of the ALK1 receptor raises a strong possibility that some of these ER-retained mutants may form heterodimeric complexes with the WT, thereby hijacking it and impairing its trafficking to the plasma membrane in a dominant-negative manner. Recently, variants of ALK1 that exhibit a normal trafficking to the plasma membrane were also proposed to exert a dominant-negative effect on the WT via forming dysfunctional heterodimers on the plasma membrane, which further impedes the 50% functionality of WT expressed by the unaffected allele [49]. These findings further consolidate the prediction that some ER-retained mutants may exhibit a dominant-negative effect on the WT protein through heterodimerization between the mutant and WT alleles.

Pulmonary arterial hypertension (PAH, # 178600) is another hereditary vascular disease associated with heterozygous mutations in *BMPR2* gene, encoding yet another type 2 receptor; bone morphogenetic protein receptor 2 [170]. Considerable research efforts have been carried out to understand the molecular mechanisms that may contribute to the wide spectrum of the disease phenotypes as well as the reduced penetrance rate, (reviewed in [171]. In an effort to understand the mechanisms by which missense mutant variants lose their functionality, we investigated the trafficking of various disease-causing variants spanning all receptor’s domains. Our findings revealed that some variants that harbor mutations at the ligand binding domain are entirely or partially trapped in the ER, ultimately leading to premature degradation through, most likely, the ERAD mechanism [15]. Retention of disease-causing missense variants of BMPR2 and the consequential defective trafficking of the receptor to the plasma membrane, which also has also been reported by others, further consolidates the implication of the ERQC mechanism in the pathogenesis of PAH [50, 172]. Furthermore, evidence of a dominant-negative effect exerted by BMPR2 missense variants on a type 1 receptor has also been reported [50]. Remarkably, there has been no exploration into whether those ER-retained mutants would exert a dominant-negative effect on WT BMPR2 expressed from the unaffected allele. Like endoglin and ALK1, BMPR2 is a homodimeric protein. This characteristic raises the possibility that heterodimerization between WT and dominant-negative ER-retained mutants may occur during the early stages of protein biogenesis in the ER, impairing its trafficking to the plasma membrane.

Loeys-Dietz Syndrome (LDS, MIM # 609192) is a rare genetic disorder inherited in an autosomal dominant manner [173]. It is characterized by multisystemic phenotypic presentations including aortic/arterial aneurysms in addition to craniofacial, osteoarticular, musculoskeletal, and cutaneous malformations [173, 174]. Up to date, mutations in six genes (TGFBR1, TGFBR2, SMAD2, SMAD3, TGFB2, and TGFB3) that encode TGFβ signaling components have been associated with LDS [174, 175]. Functional assays have demonstrated that mutated TGFBR1 interfere with the endogenously expressed WT receptor, reducing its activity in a dominant-negative manner [52]. These findings open the doors for further investigation into the molecular mechanisms that lead to the loss of function of these major components in the signaling pathway. A possible dominant-negative effect exerted by the mutant allele on the WT may also represent logical contributor to an aggravated haploinsufficiency state in LDS. Furthermore, considering that LDS is associated with several mutant variants of components functioning along the same signaling pathway, it is worthwhile to investigate whether some of these mutant components might interfere with the function of multiple components within the signaling pathway, potentially contributing to the observed heterogeneity in LDS phenotypes.

It is crucial to highlight that the majority of TGFβ signaling pathways involve dimeric proteins, whether secretory or membrane-bound. As illustrated in a prior review, through an extensive literature search, we have shown that these proteins are implicated in around 25 monogenic human diseases [109]. However, the disease mechanisms of these conditions remain underexplored in terms of possible implication of ERQC mechanism and also the potential existence of dominant-negative effects. Further investigation into these aspects is warranted to enhance our understanding of the pathogenesis associated with TGFβ signaling-related monogenic diseases.

Long QT syndromes (LQTS) are a group of autosomal inherited arrhythmogenic disorders characterized by abnormal cardiac activity presented by prolonged QT intervals, leading to a type of arrhythmia known as torsades de pointes [176]. Irregularities in the heartbeat have the potential to result in fainting, seizures, or sudden cardiac arrest. LQTS is classified into three primary types based on the causative genes: LQTS1, MIM # 192500, LQST2, MIM # 613688, and LQTS3, MIM # 603830 encoded by the genes (*KCNQ1*), (*KCNH2*) and (*SCN5A*), respectively. These genes encode ion channels essential for cardiac repolarization [177]. Nonetheless, each type has distinct triggers, clinical manifestations, severity and penetrance profile, suggesting variable molecular mechanisms involved, in addition to environmental factors, age and gender [178]. LQTS2 is associated with *KCNH2*, a gene that encodes the voltage-gated K^+^ channel α-subunit (Kv11.1), which function as tetrameric complex that consists of four Kv11.1 α-subunit [179].

Ficker and colleagues reported through immunoprecipitation analysis that Kv11.1 disease-causing variants R752W and G601S show defective trafficking, evidenced by their strong association with molecular chaperones Hsp90 and Hsp70 in the ER. Defective trafficking results in ER-retention of misfolded Kv11.1variants, followed by premature degradation through the ERAD mechanism. Conversely, the non-functional G628S variant displayed transient associations with the molecular chaperones before being released to the plasma membrane, similar to WT Kv11.1 [54]. Characterizing the physiological properties of Kv11.1 disease-causing variants harboring the missense mutation (E637K) situated in the pore-S6 loop of the channel, using a Xenopus oocyte heterologous expression system, revealed intriguing findings. Coexpression of WT (WT) and E637K variants resulted in a peak tail current significantly lower than the current peaks anticipated from the WT alone. These findings suggest that this mutation exerts a dominant-negative effect on the WT Kv11.1 channel and highlights the significance of the pore-S6 loop in the channel's function [180, 181]. Therefore, if a dominant-negative effect can occur on the plasma membrane through the formation of the WT/mutant tetrameric complexes, then it follows logically that ER-retained mutants may also form similar tetrameric complexes in the ER, that impede the trafficking of the WT ion channel to the plasma membrane, employing a similar dominant-negative mechanism.

### Neurological disorders

The spectrum of molecular mechanisms underlying monogenic neurological disorders with autosomal dominant inheritance is diverse, reflecting the wide array of genes and protein variants involved. Nonetheless, protein misfolding and aggregation are recurrent themes, leading to cellular death and permanent neurological dysfunction. Phenotypic traits can be further aggravated by dominant-negative variants that interfere with the remaining 50% functionality of the WT protein.

Heterozygous mutations in the GABAA receptor gamma2 subunit encoded by the gene *GABRG2* have been associated with generalized epilepsy with febrile seizures plus (GEFS + , MIM # 607681) [59, 182]. The GABAA receptor, a pentameric ligand-gated ion channels serving as a receptor for the inhibitory neurotransmitter gamma-aminobutyric acid (GABA), is typically composed of two α subunits, two β subunits, and one γ2 subunit [183]. The molecular mechanisms associated with disease-causing variants were identified to involve haploinsufficiency attributable to the mutant allele, along with the exertion of a dominant-negative effect by the mutated γ2 subunit. Kang and colleagues have demonstrated, using in vitro cellular models, that the pathogenic variant γ2(Q351X) associated with GEFS exhibits defective trafficking to the plasma membrane, leading to proteasomal degradation through ERAD. Additionally, γ2(Q351X) was shown to exert a dominant-negative effect on WT receptors, reducing their assembly, trafficking, and surface expression ( 59). This effect likely arises from the oligomerization of mutant and other WT subunits that form the full pentameric receptor complex, resulting in ER retention of both WT and mutant subunits followed by premature degradation through ERAD. Pulse-chase experiments revealed that coexpression of the γ2 subunit mutation, Q351X, with other WT subunits reduced GABAA function to a level lower than the predicted 50% level for heterozygous carriers. Therefore, the combined mechanism of ER retention and dominant-negative effect exerted by the mutant γ2 subunit on partnering subunits is predicted to be the most likely mechanism for the manifestation of GEFS + associated with this mutation.

A combination of haploinsufficiency and dominant-negative effect mechanisms has also been identified as the underlying mechanism for severe cases of Neurofibromatosis type 1 (NF1, MIM # 162200), a rare genetic disorder characterized by the development of tumors on nerve tissues throughout the body [61]. Neurofibromin, encoded by NF1 gene, is a tumor suppressor and a GTPase activating proteins that regulates RAS signaling cascade in various cell types including neuronal cells [184]. In invitro cellular models, it has been demonstrated that misfolded neurofibromins, encoded by variants in codons 844 to 848, exert a destabilizing effect on the WT protein through protein dimerization in the ER. Heterodimeric complexes are recognized by the ERQC mechanism and marked for degradation via ERAD mechanism. This interaction results in a complete reduction of neurofibromin levels, surpassing the threshold observed in haploinsufficiency alone [185].

In genetic disorders, dominant-negative effect might also occur when a mutated protein disrupts the function of another protein it unexpectedly interacts with, leading to the manifestation of disease symptoms. This phenomenon is best demonstrated by missense variants of voltage-gated potassium channel (Kv3.3) associated with autosomal dominant spinocerebellar ataxias 13 (SCA13, MIM #176264), encoded by the gene *KCNC3* [186]. These misfolded variants display impaired trafficking, resulting in entrapment within endosomal vesicles and a failure to reach their designated functional location at the plasma membrane [60]. Intriguingly, they also intracellularly engage with the human epidermal growth factor receptor (EGFR) through an unknown mechanism, implying a pivotal role for EGFR in cerebellum development and, consequently, highlighting its involvement in the pathology of SCA13.

DYT1, also known as early onset torsion dystonia, is an autosomal dominant neurological disease characterized by involuntary muscle contractions (dystonic movements) affecting several parts of the body, resulting in severe disability [187]. The disease typically begins in childhood or adolescence and represents the most common and severe type of dystonias. DYT1 dystonia is caused by a specific mutation in the *TOR1A* gene, which involves the deletion of three base pairs (GAG), resulting in the loss of a single glutamic acid residue in the torsinA protein at position 302 (ΔE-torsinA) [63]. The torsinA protein is an ER-resident glycoprotein and a member of the AAA + (ATPases Associated with diverse cellular Activities) protein family that play key roles in cellular functions related to protein folding, trafficking, and/or degradation [188]. Oligomerization is a conserved feature of members of the AAA^+^ family of ATPases, however, unlike WT-torsinA, mutant ΔE-torsinA is mislocalized and forms perinuclear aggregates [63]. Furthermore, through coimmunoprecipitation assays, it has been shown that WT torsinA interacts with the mutant protein, resulting in a dominant-negative effect by sequestering WT protein to the perinuclear region, where they form multimeric protein complexes [64]. The number of remaining functional WT-torsinA multimers would depend on the expression ratio of WT-torsinA: ΔE-torsinA. Interestingly, investigations into the degradation pathways of WT and mutant ΔE torsinA proteins have revealed divergent degradation pathways for each. WT-torsinA was found to degrade through autophagy, while ΔE-torsinA, which exhibited a significantly shorter half-life, was selectively and efficiently degraded via the proteasome through ERAD, rendering it a potential target for interventional rescue [64].

### Eye disorders

Retinitis pigmentosa (RP, MIM # 613731) is a genetic degenerative eye disorder that is associated with mutations in numerous genes, which contribute to the heterogeneity of the disease. Mutations in rhodopsin gene (*RHO*) have been identified as one of the most common causes of autosomal dominant PR [189]. Rhodopsin is a light-sensitive receptor protein that plays a crucial role in the conversion of light signals into electrical signals in rod photoreceptor cells of the retina. *RHO* mutations have been categorized into seven categories according to their effect on the protein’s structure and function, reviewed in [67]. Rhodopsin has a high ability to form dimeric and higher order oligomers which is believed to play a key role in the photoreceptor function [190]. The most common disease-causing misfolded rhodopsin variants P23H and K296E were found to form ER-retained aggregates followed by proteasomal degradation. But if degradation was not complete, mutant rhodopsin can accumulate in photoreceptor cells, leading to cell degeneration and contributing to the development of the disease phenotypes [66]. In addition, It was demonstrated using fluorescence microscopy and immunoblot analysis that co-expression of variants P23H or G188R, together with the WT rhodopsin has resulted in the entrapment of WT rhodopsin in the ER. Misfolded dimers aggregate with the WT Rhodopsin exerting a dominant-negative effect through hetero-oligomerization [68, 69].

Primary open angle glaucoma (POAG, # 137750), has also been identified as a leading cause for irreversible blindness due to damaged optic nerve [191]. Mutations in the myocilin gene (*MYOC*) have been associated with the disorder in several families with early onset of visual impairment. Total loss of myocilin in samples of patients harboring heterozygous mutations has been attributed to a combinational mechanism of haploinsufficiency and dominant-negative effects exerted by the entrapped mutant myocilin on the WT expressed from the functional allele [72].

Furthermore, Wolfram syndrome (WS, MIM # 222300) is a multisystemic syndrome that is characterized primarily by diabetes mellitus, optic atrophy which represent a major feature affecting nearly all reported patients, in addition to sensorineural deafness, neurodegeneration and psychological imbalances [192]. The autosomal dominant form of the disease is caused by heterozygous mutations in the *WFS1* gene that encodes wolframin, a transmembrane protein that regulates calcium homeostasis within the ER. In contrast to homozygous mutants, It has been reported by many that some heterozygous misfolded wolframin exhibit a dominant-negative effect on the WT as it aggregates in the ER lumen causing ER stress and cellular toxicity [75, 193, 194].

### Serpinopathies

Serpins are a large group of serine protease inhibitors that play a key role in regulating proteases activities across various organs [195]. Mutations in these genes result in the aggregation of mutant proteins, inducing cellular dysfunction and giving rise to a spectrum of monogenic disorders collectively termed serpinopathies. Serpins are inherently unstable, and this is mainly due to their mechanism of action, as they alternate between folded and unfolded state in order to perform their inhibitory function. Mutations that lead to unfolded proteins destabilize this fine balance and promote protein aggregate formation [196]. Heterozygous mutations in *SERPINC1, SERPINA1and SERPING1* have been associated with autosomal dominant antithrombin deficiency (MIM #107300), Alpha-1-antitrypsin deficiency (A1ATD, MIM # 613490) and hereditary angioedema type 1 (HAE1,MIM # 106100), respectively. Dominant-negative variants of these three genes have been associated with the formation of mutant/WT protein aggregates in the ER, resulting in significant reduced plasma levels of the respective proteins and correlating with severe disease phenotypes [78, 79, 81].

## Therapeutic challenges and emerging strategies in dominant-negative disorders

**Fig. 3:**
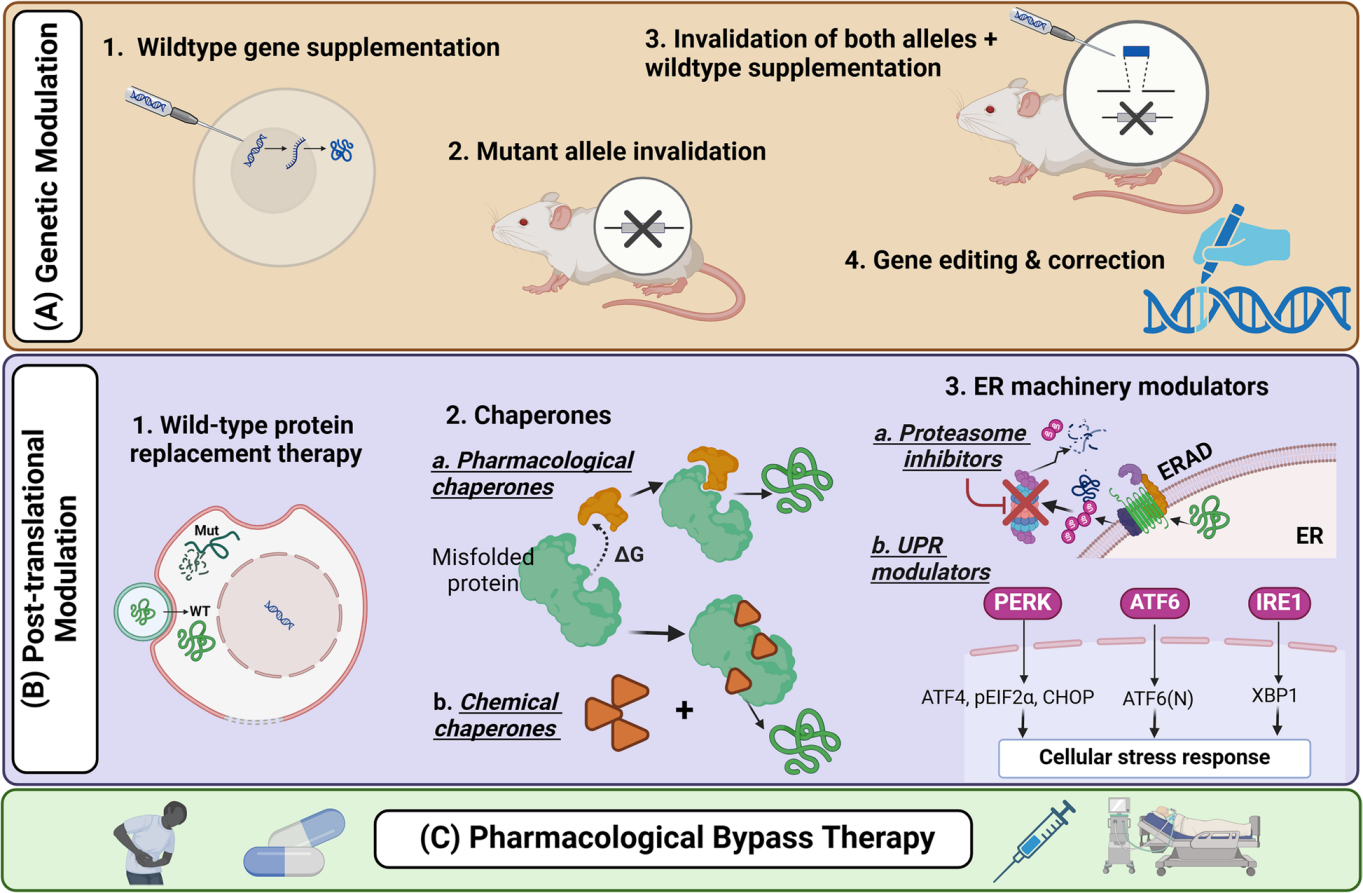
Therapeutic Strategies for Dominant-negative Disorders. **A** Genetic modulation directly tackles the faulty gene through diverse techniques by introducing a functional copy, eliminating the mutated allele, combining both approaches, or even editing the mutation itself using advanced tools like CRISPR/Cas9 and base editing techniques **B**) Post-translational modulation mainly targets correcting the underlying folding or structural deformity through chemical and pharmacological chaperones, or through directly manipulating specific components of the ER quality control machinery. For some diseases, recombinant proteins are administered to compensate for the loss of the WT protein **C**) Pharmacological bypass therapy uses drugs or different pharmacological compounds to mimic the function of the WT protein or compensate for its absence, even without repairing the gene, to relieve the underlying clinical symptoms by targeting downstream pathways affected by the mutation

Treating conditions that include dominant-negative effect as a contributing mechanism can be a difficult task for medical professionals, as it presents a significant therapeutic challenge. Simply increasing protein levels is not always an effective strategy because as highlighted earlier, the mutant protein may interfere with the function of its WT counterpart [197]. This combination of the "poisoning" effect and potential ER retention and aggregation, resulting from the ER machinery involvement, requires alternative therapeutic approaches. Strategies designed to address the pathological consequences of some dominant-negative mutations fall into three main categories, utilizing diverse tools and techniques summarized in (Fig. 3).

Therapeutic interventions aimed at counteracting dominant-negative effects may show promise for disorders characterized by ER-retention dominant-negative mechanisms. However, their efficacy depends on factors such as the nature of the disorder, the specific genetic mutation involved, and the mechanism of action of the intervention.

### Genetic modulation

The straightforward approach in genetic modulation that comes to mind is to introduce a functional copy of the defective gene into the diseased cell. Therapeutic approaches aimed at increasing the amount of WT protein in autosomal dominant diseases caused by dominant-negative variants can also be effective for variants exhibiting the combinational mechanism of ER retention and dominant-negative effects. The underlying principle is the same: enhancing the availability of functional WT protein to compensate for the defective mutant protein. By boosting the levels of WT protein, these therapies can mitigate the impact of the dominant-negative mutant, regardless of whether the mutation leads to ER retention or other misfolding issues. This strategy holds promise for improving cellular function and alleviating disease symptoms across a range of genetic disorders with similar pathogenic mechanisms. In RP disorder for example, increasing WT rhodopsin to three-fold its normal level in the eyes of transgenic mice carrying the P23H ER-retained/dominant-negative variant has been shown to protects the retina, suggesting gene therapies that carefully boost rhodopsin levels could alleviate such diseases [198]. Delivering a normal copy of the *RHO* gene via adeno-associated virus serotype 5 (AAV) vector prevented retinal degeneration in P23H transgenic mice through the increased expression of WT rhodopsin. This aims to increase the WT to mutant protein ratio, potentially out-competing the mutant protein and subsequently restoring some function [199]. Similarly, the introduction of a WT copy of the GABRG2 gene into transgenic mice carrying the dominant-negative Q390X variant (Gabrg2 + /Q390X) associated with Dravet syndrome (epileptic encephalopathy) significantly rescued their underlying seizures [58]. However, mutated proteins with dominant-negative effects may still exert detrimental effects even with increased WT protein expression. Therefore, treating such conditions might necessitates targeting the mutant allele at the DNA or RNA levels [200]. AAV vectors have been employed to selectively disrupt the expression of the dominant-negative allele in the *COL1A1* gene through the insertion of a neomycin resistance cassette to the first exon of the gene in mesenchymal stem cells (MSCs) derived from patients presented with OI, successfully demonstrating targeted gene modification in adult human stem cells [201]. Alternatively, various methodologies can be used to silence the mutated allele at the transcription level via antisense oligodeoxyribonucleotides (ODNs), short interfering RNA (siRNA), and hammerhead ribozymes. In this context, highly specific siRNA has been designed to selectively suppress the mutant torsin A protein, the primary causative factor in the most common form of primary generalized dystonia [202]. It efficiently suppressed the mutant torsin A in cells mimicking the heterozygous state without affecting the WT allele.

Given the limitations of each approach individually in effectively bypassing the underlying defect, especially for cells sensitive to haploinsufficiency, their synergistic application emerges as a viable solution. Dotzler SM and colleagues presented a therapeutic solution by incorporating a suppression-and-replacement genetic approach for LQT1 syndrome that utilizes a two-component strategy [203]. The first component is based on the use of a short hairpin RNA (shRNA) to specifically suppress the expression of the patient's endogenous, mutated *KCNQ1* gene. Secondly, the introduction of a codon-altered, shRNA-immune copy of *KCNQ1*, thereby achieving functional replacement of the defective allele. This dual-pronged approach demonstrates promising preclinical efficacy, as evidenced by the successful restoration of normal function in induced pluripotent stem cell-derived cardiomyocytes harboring diverse LQT1-causing *KCNQ1* variants. Furthermore, it represents a feasible therapeutic strategy that can effectively address the double whammy of ER-retention and dominant negative effects by specifically silencing the mutant allele, which prevents the production of the defective protein, thereby reducing its detrimental interactions with the WT protein. Additionally, it helps in mitigating issues related to ER retention, as fewer mutant proteins are available to be retained in the ER. Consequently, more WT proteins can function correctly, improving cellular function and potentially ameliorate disease symptoms.

Fueled by the discovery of CRISPR/Cas9 technology, genetic tools have been developed to directly correct the disease-causing variant in different genetic diseases [204]. CRISPR/Cas9-mediated gene correction has been implemented in OI patients’ derived iPSCs which were differentiated to Osteoblast cells with recovered type I collagen levels [205]. Besides that, Huang et al. leveraged cutting-edge base editing technology to precisely repair a disease-causing mutation (FBN1; T7498C) in MFS, demonstrating the potential of this approach for gene therapy in MFS and other genetic disorders [26]. Unlike CRISPR/Cas9 base editing technology, the proposed technology is more precise with minimal risk of unintended mutations as it does not require the generation of a double-strand break to correct the intended nucleotide [206].

### Post-translational modulation

Instead of genetically manipulating the affected gene, administration of exogenous WT proteins through protein replacement therapy has shown successful outcomes in a few diseases with dominant-negative pathophysiology. Although not universally applicable to dominant-negative disorders, the clinical success of recombinant intravenous (IV) C1INH formulations in hereditary angioedema patients underscores the therapeutic potential of protein replacement therapy for this class of diseases [207]. It demonstrates its ability to mitigate the pathological protein misfolding and abnormal ER aggregation caused by the underlying heterozygous *SERPING1* variations. It's important to recognize that protein replacement therapy, despite its success in some dominant-negative diseases, may not be feasible for all due to technological limitations and individual patient factors.

Interventions that promote proper folding and prevent aggregation of the mutated protein show promise in rescuing the WT protein from ER entrapment. Pharmacological chaperones (Pcs) have been used in this context to specifically bind to the mutated protein promoting its proper folding and stabilization which will subsequently prevent its retention and premature degradation along with its WT counterpart [208]. Therefore, the application of Pcs presents a compelling strategy for targeting the combinational mechanism of dominant negative effects exerted by ER-retained mutant variants. Several studies have demonstrated the potential of retinoid analogs to act as specific PC compounds for the P23H mutation in rhodopsin, which causes RP [209]. These chaperones enhance the folding of the mutant protein and reduce its dominant-negative effect on the processing of the WT form [69]. Similarly, the IN3 PC compound corrects folding errors of several GnRH receptor (GnRHR) mutants; causative of hypogonadotropic hypogonadism, and promotes its correct intracellular trafficking along with its interacting WT subunits [210]. Unlike the targeted approach of PCs, chemical chaperones exhibit a broad-spectrum effect stabilizing various proteins and preventing aggregation in a non-specific manner [211]. For example, 4-phenylbutyrate (4-PBA) is a clinically approved medication for urea cycle disorders that showed its potential to prevent P23H rhodopsin aggregation and reduce the associated ER stress in RP [67]. In addition, protein rescue may also involve targeting specific components of ERAD and the ER machinery. Proteasomal inhibitors such as MG-132, MG-115, lactacystin, or proteasome inhibitor I prevented the premature degradation of ER-tagged caveolin-3 mutants, rescuing their interacting WT forms in a LGMD-1C cellular model [34]. Moreover, targeting the abberent activation of the UPR pathway due to ER stress in cells with accumulated misfolded proteins may offer a potential therapeutic approach in dominant-negative diseases. In a mouse model of RP, knocking out ATF4 in mice expressing the dominant-negative T17M rhodopsin mutation halted retinal degeneration. Blocking ATF4 expression lead to the downregulation of multiple UPR components like pEIF2α, ATF6, and CHOP, ultimately blocking the activation of cell death pathways [212]. Overall, addressing distinct elements within the ER machinery, aiming to mitigate the dominant-negative consequences caused by misfolded proteins and restore the WT from the underlying damage, signifies an innovative and promising frontier where cell biology intersects with medicine.

### Pharmacological bypass therapy

Besides, several therapeutic interventions have been utilized to bypass the need to directly manipulate the underlying defect with various agents or pharmacological medications, often referred to as phenotypic correctors, that resemble the downstream effects of the WT protein. In ISS therapy, long-term growth hormone (GH) treatment can increase the height in childhood and adult life of familial and nonfamilial ISS cases including patients carrying heterozygous variants in the NPR2 gene showing dominant-negative effects [213]. Despite the response variability towards GH therapy, several NPR2 cases showed promising responses in height correction, especially with earlier (before puberty) and long-term administration [32, 214]. On the other hand, myoblast cultures derived from patients with UCMD, caused by mutations in COL6A1, COL6A2, or COL6A3 genes, displayed increased cellular apoptosis. Oral treatment with cyclosporine A (an immunosuppressive drug; CsA) for one month significantly reduced apoptosis through the normalization of the mitochondrial membrane potential of the tested muscle cells [215]. The overall conclusion from this pilot study is that long-term CsA treatment influences myofiber regeneration and ameliorates muscle cell performance in treated patients. HAE1, characterized by uncontrolled plasma kallikrein due to C1INH deficiency even with heterozygous carriers, shows enhanced treatment response to drugs inhibiting kallikrein, leading to significant clinical improvement [207].

## Future perspectives and conclusions

The dominant-negative effects exerted by mutant proteins on either their WT allele or interacting partners represent a major mechanism underlying various autosomal dominant genetic diseases and may contribute significantly to their wide spectrum of phenotypic clinical manifestations. Furthermore, an additional combined mechanism emerges when ER-retained mutant proteins form mixed complexes with WT counterparts or multi-subunit partners, resulting in the mis-localization and premature degradation of these WT partners. As a consequence, an additional loss of functional protein occurs, further compromising cellular function and exacerbating disease phenotypes. Thus, the dual additive impact of the dominant-negative effects and ERAD-mediated degradation is playing a pivotal role in the complexity of disease pathogenesis in numerous autosomal genetic disorders. Notably and surprisingly, this specific and highly damaging combinatorial mechanism remains relatively understudied and underappreciated in the field. This review represents an initial effort to illuminate and highlight this aspect of research, presenting significant potential for elucidating the factors influencing variant-associated phenotypic variability and detailed disease pathogenesis in numerous conditions. By highlighting these complex interactions, this review aims to promote further exploration and potentially uncover novel avenues for understanding and addressing mechanisms underlying autosomal dominant diseases. Furthermore, understanding these intricate mechanisms may offer insights into potential novel therapeutic strategies aimed at mitigating clinical presentations in these diseases including ameliorating their severity.

## Data Availability

All dataset was incorporated in this manuscript.

## Abbreviations

4-PBA: 4-Phenylbutyrate
ATF6: Activating transcription factor 6
BMD: Bethlem myopathy disorder
CFTR: Cystic fibrosis transmembrane conductance regulator
CNP: C-type natriuretic peptide
CNX/CRT: Calnexin/calreticulin
DEB: Dystrophic epidermolysis bullosa
ECM: Extracellular matrix
EDS: Ehlers-Danlos syndrome
EGFR: Epidermal growth factor receptor
ER: Endoplasmic reticulum
ERAD: ER-associated protein degradation
ERQC: ER quality control
GABA: Gamma-aminobutyric acid
GEFS +: Febrile seizures plus
GH: Growth hormone
GnRHR: GnRH receptor
HHT1: Hereditary haemorrhagic telangiectasia type 1
HHT2: Hereditary haemorrhagic telangiectasia type 2
Hsp: Heat shock proteins
IRE1: Inositol requiring enzyme 1
ISS: Idiopathic short stature
IV: Intravenous
LDS: Loeys-Dietz Syndrome
LGMD: Limb-girdle muscular dystrophy
LQTS: LQT syndromes
MFS: Marfan syndrome
MSCs: Mesenchymal stem cells
MYOC: Myocilin gene
NMD: Nonsense-mediated decay
NF1: Neurofibromatosis Type 1
ODNs: Oligodeoxyribo nucleotides
OI: Osteogenesis imperfecta
PAH: Pulmonary arterial hypertension
Pcs: Pharmacological chaperones
PERK: Protein kinase RNA like endoplasmic reticulum kinase
POAG: Primary open angle glaucoma
RHO: Rhodopsin gene
RP: Retinitis pigmentosa
shRNA: Short hairpin RNA
siRNA: Short interfering RNA
STL1: Stickler syndrome type1
STL2: Stickler syndrome type 2
TGFβ: Transforming growth factor beta
UCMD: Ullrich congenital muscular dystrophy
UGGT: UDP-glucose: glycoprotein glucosyltransferase
UPR: Unfolded protein response
VWD: Von Willebrand disease
VWF: Von Willebrand factor
WS: Wolfram syndrome
WT: Wild-type
